# Biomechanical design of titanium-PEEK combined fusion cage based on PLIF surgical model

**DOI:** 10.3389/fbioe.2025.1671068

**Published:** 2025-10-14

**Authors:** Chunsheng Liu, Yutang Xie, Xinrui Wu, Yanqin Wang, Yanru Xue, Pengcui Li, Wangping Duan, Xiaochun Wei, Weiyi Chen, Jinzhu Yin, Kai Zhang, Meng Zhang, Xiaogang Wu, Liming He

**Affiliations:** ^1^ College of Artificial Intelligence, Taiyuan University of Technology, Taiyuan, China; ^2^ Shanxi Key Laboratory of Bone and Soft Tissue Injury Repair, Department of Orthopaedics, The Second Hospital of Shanxi Medical University, Taiyuan, China; ^3^ Sinopharm Tongmei General Hospital, Datong, China; ^4^ Huajin Orthopaedic Hospital, Taiyuan, China; ^5^ Shanxi Bethune Hospital, Shanxi Academy of Medical Sciences, Tongji Shanxi Hospital, Third Hospital of Shanxi Medical University, Taiyuan, China

**Keywords:** lumbar fusion, biomechanical analysis, finite element method, stress shielding, fusion device design

## Abstract

Fusion devices play a critical role in lumbar fusion surgery. Titanium alloy fusion devices offer good biocompatibility and stability, but their mechanical properties far exceed those of bone, leading to stress shielding effects after implantation, which can reduce spinal fusion rates and cause endplate collapse. On the other hand, fusion devices made of polyether ether ketone (PEEK), which has a lower elastic modulus, are not conducive to bone ingrowth and fusion stability due to their material properties. Personalized fusion devices that can precisely adapt to a patient’s physiological condition are not widely used due to their lengthy design cycle. This study proposes an optimized design method based on a titanium alloy-PEEK composite structure. By constructing three composite structure models—PEEK core and Ti frame (square hole type, circular hole type, plate type)—and combining finite element compression simulation with machine learning algorithms, the structural parameters are intelligently optimized. The machine learning algorithm used in this study is Back Propagation Neural Network. The aim of this study is to match the equivalent elastic modulus of the fusion device with that of cortical bone. The three optimized fusion devices, along with the Ti fusion device and PEEK fusion device as control groups, were implanted into a traditional PLIF postoperative model for static and transient dynamic analysis. The biomechanical responses of the lumbar spine at various locations after implantation of the five fusion devices were analyzed and compared. The results indicate that all three optimized fusion devices effectively reduce the risk of device settlement, thereby mitigating stress shielding effects, improving fusion rates, and enhancing postoperative lumbar stability. Among them, the circular hole inner core fusion device (M2) demonstrated the best overall performance. The peak von Mises stress of L4 lower endplate and L5 upper endplate in M2 model were 54.2% and 27.7% respectively lower than those in Ti fusion device. Compared with Ti fusion device, the strain energy of M2 model increased by 49.7%. The development framework of this study which integrated “finite element simulation-machine learning-postoperative model biomechanical validation and evaluation” can effectively reduce the design cycle and cost of personalized orthopedic implants.

## 1 Introduction

Posterior lumbar interbody fusion (PLIF) surgery is widely recognized as the gold standard for treating lumbar degenerative diseases due to its high fusion rates and postoperative stability. However, challenges such as device displacement and endplate stress remain significant concerns (Fan et al., 2021). However, PLIF surgery also carries risks of epidural hematoma and insufficient pressure, which may lead to fusion device displacement. Excessive endplate stress can cause fusion device settlement and damage to lumbar spinal structures.

Spinal fusion surgery emerged in the early 20th century, primarily using autologous bone grafting to replace the intervertebral disc in the surgical segment and maintain spinal structural stability. It was not until the early 1980s that O’Brien et al. pioneered a combined approach using cortical bone from the femoral ring and cancellous bone from the autologous graft to promote post-operative fusion (Hasler, 2013). While these early bone grafting methods partially met the needs for post-operative fusion, their insufficient stability prevented them from being used alone for spinal fixation. The introduction of titanium alloy fusion devices marked a significant milestone in the development of lumbar fusion devices. In 1992, Bagby and Kuslich first demonstrated the safety and efficacy of titanium alloy fusion devices in clinical trials. This titanium alloy fusion device, known as the BAK intervertebral fusion device, features a “hollow, porous, square-threaded, slightly conical, cylindrical” structure, providing higher postoperative stability (Wellington et al., 2022). In a multicenter prospective clinical trial, the BAK intervertebral fusion device achieved a fusion rate of 91% in both single-segment and double-segment lumbar fusion (Lee et al., 2024). Titanium alloy quickly became the material of choice for lumbar fusion devices due to its excellent biocompatibility and mechanical stability.

With the advancement of research in spinal fusion surgery, the materials and structures of fusion devices have also been continuously optimized. In the 1990s, PEEK was introduced as a new material in spinal fusion surgery (Verma et al., 2021). PEEK is a high-performance thermoplastic polymer with an elastic modulus similar to that of cancellous bone (approximately 3.8 GPa), which reduces the risk of fusion device settlement and fusion failure while avoiding the stress shielding effect on the lumbar spine structure postoperatively (Campbell et al., 2020). Additionally, its excellent radiographic transparency aids fusion assessment. However, PEEK’s surface is prone to biofilm formation, which inhibits bone-implant osseointegration—a limitation that still needs better solutions. To address this limitation, researchers have explored surface coating technologies (Hasegawa et al., 2020). Coating the PEEK surface with titanium via plasma spraying significantly improves osseointegration at the bone-implant interface. Furthermore, coating the titanium alloy surface with bioactive substances such as hydroxyapatite via electrophoretic deposition technology can further enhance osseointegration (Tan et al., 2021). The application of these surface treatment technologies has led to significant progress in improving biocompatibility and promoting osseointegration in fusion devices.

At the beginning of the 21st century, 3D printing technology—especially selective laser sintering (SLS) provided a new direction for lumbar fusion devices, enabling the manufacture of titanium alloy devices with complex internal structures (Kafle et al., 2021). The 2004 launch of metal-powder-melting SLS printers further promoted their commercial production. By 2015, a growing number of clinical trials utilizing 3D-printed titanium alloy fusion devices began to emerge (Witowski et al., 2018; Al-dulimi et al., 2021). In 2017, the U.S. Food and Drug Administration (FDA) issued guidelines for the design, manufacturing, and testing of 3D-printed medical devices (Seifi et al., 2017). The advantages of 3D-printed titanium alloy fusion devices lie in their ability to be customized according to the patient’s specific anatomical structure, thereby improving the device’s fit and stability. Additionally, 3D-printed titanium alloy fusion devices can further optimize their mechanical properties and biocompatibility by adjusting the porosity and density of the device. Studies have shown that 3D-printed porous titanium alloy fusion devices outperform traditional PEEK fusion devices and titanium alloy fusion devices in reducing sinking rates, improving clinical outcomes, and promoting osseointegration (Zhang et al., 2020). A retrospective study of 113 patients found that patients using 3D-printed titanium alloy fusion devices experienced fewer cases of severe fusion device sinking compared to those using PEEK fusion devices (Makino et al., 2021).

In this study, we propose three new design schemes for composite material combinations suitable for lumbar interbody fusion surgery. The designed fusion devices achieve an equivalent elastic modulus close to that of the vertebral cortical bone, while the fusion device-bone endplate interface uses titanium alloy materials as much as possible. This provides sufficient structural support strength and promotes osseointegration for the lumbar spine postoperatively while avoiding the stress shielding effect caused by excessive fusion device rigidity. Finally, finite element methods were used to conduct a comprehensive analysis of the biomechanical response of the PLIF postoperative model under static loads and whole body vibration loads.

## 2 Methods

### 2.1 Lumbar intact model establishment

The studies involving humans were approved by the institutional review board (IRB) of the Shanxi Bethune Hospital (YXLL-2022-147). CT scan images of the spine (Dicom 3.0 format) were obtained from a healthy volunteer. The tomographic images were imported into Mimics Research 21.0 (Materialize, Leuven, Belgium), and the CT files were processed using functions such as threshold segmentation to extract the three-dimensional surface of the lumbar spine at the L4-L5 segment. The exported three-dimensional surface was imported into Geomagic Studio 2021 (3D Systems, Ltd., United States) for surface smoothing. In the software, the surface segments were fitted to construct a three-dimensional solid model. Using Siemens NX 10.0 (Siemens, Germany) to assemble the model and establish structures such as intervertebral discs and facet joint cartilage. The skeletal mesh model and ligament structures were constructed using Hypermesh (Altair Engineering, Troy, Michigan, United States). Considering, the biomechanical property that ligaments withstand tensile loads but not compressive loads, six ligament structures were established using truss elements (Murphy and Brown, 2021), including the anterior longitudinal ligament (ALL), posterior longitudinal ligament (PLL), yellow ligament (LF), capsular ligament (CL), intertransverse ligament (ITL), interspinous ligament (ISL), and supraspinous ligament (SSL) (Polikeit et al., 2003).

**FIGURE 1:**
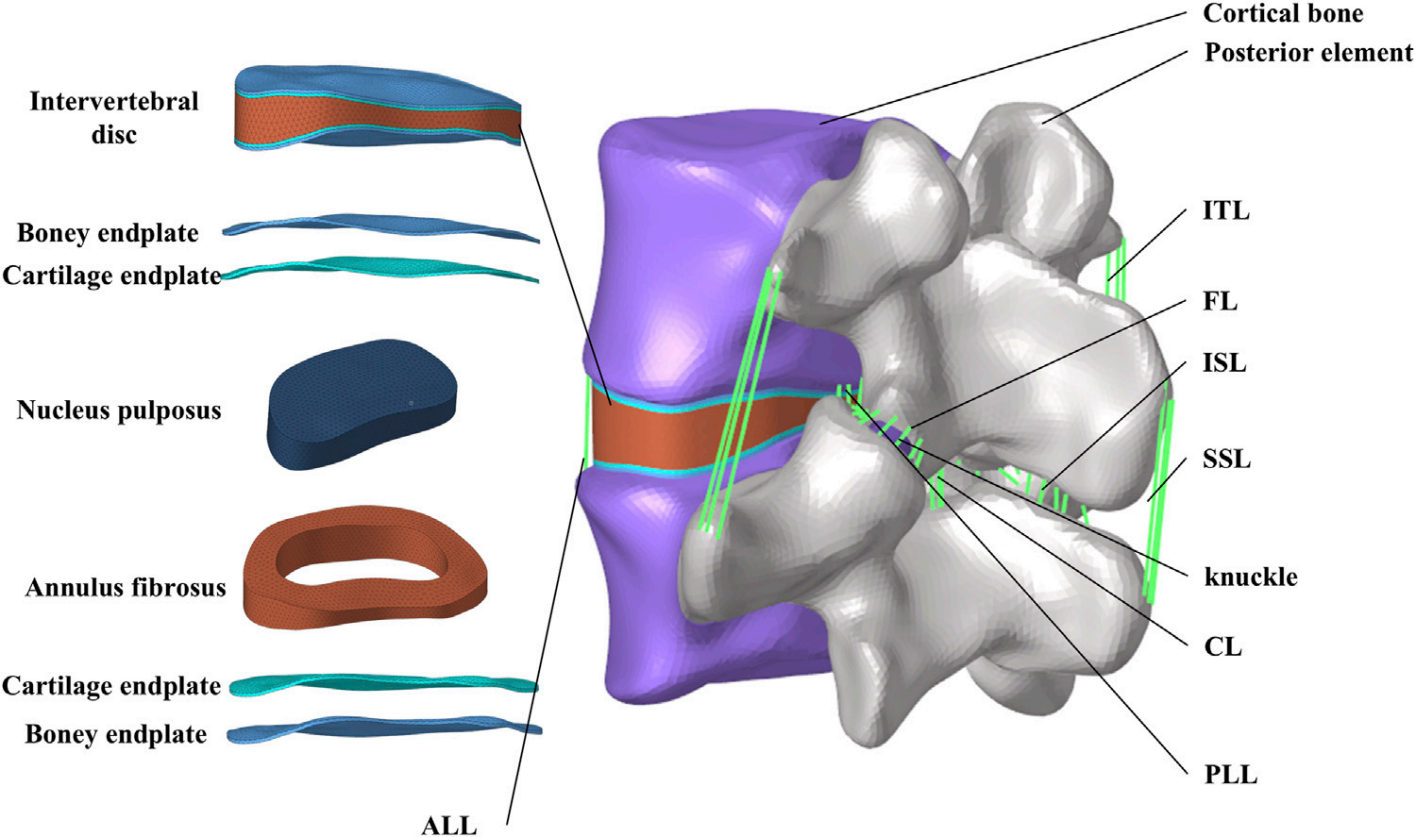
FE model of the L4-L5 lumbar vertebrae.

Abaqus software (Hibbitt, Karlsson, and Sorensen, Inc., Providence, Rhode Island, United States) was used to define material properties, set boundary conditions, and perform finite element analysis. Figure 1 shows the finite element model of the complete L4-L5 lumbar vertebrae. The thicknesses of the cortical endplates and cartilaginous endplates are 0.5 mm and 0.5 mm, respectively. The intervertebral disc is divided into the nucleus pulposus and the annulus fibrosus. The nucleus pulposus accounts for 30%–40% of the total volume of the intervertebral disc. The nucleus pulposus and annulus fibrosus are simulated using Mooney-Rivlin hyperelastic material. Between the superior and inferior articular processes, there is articular cartilage that reduces joint friction, with a friction coefficient of 0.1 between the cartilage and the articular processes. The material properties of each vertebral structure are detailed in Table 1 (Ayturk and Puttlitz, 2011; Yan et al., 2023).

**TABLE 1:** Material properties of finite element model.

Materials	Elastic modulus (MPa)	Poisson’s ratio	Unit type	Cross-sectional area (mm^2^)/Thicknesses (mm)	Mass density (kg/mm^3^)
Cortical bone	12000	0.3	C3D4	1	1.7e-6
Cancellous bone	100	0.2	C3D4	—	1.1e-6
Back-end architecture	3500	0.3	C3D4	—	1.4e-6
Bony endplate	2000	0.2	C3D4	0.5	1.2e-6
Cartilaginous endplate	24	0.25	C3D4	0.5	—
Annulus fibrosus	MooneyRivlin C_10_ = 0.18 C_01_ = 0.045	—	C3D8RH	—	1.02e-6
Nucleus pulposus	MooneyRivlin C_10_ = 0.12 C_01_ = 0.03	—	C3D8RH	—	1.05e-6
Articular cartilage	10	0.3	C3D4	—	1.2e-6
Polyether ether ketone (PEEK)	3500	0.3	C3D4	—	1.32e-6
Fixation systems	113000	0.3	C3D4	—	4.5e-6
ALL	7.8	0.3	T3D2	75.9	1.0e-6
PLL	1	0.3	T3D2	14.4	1.0e-6
FL	1.5	0.3	T3D2	40	1.0e-6
ITL	10	0.3	T3D2	3.6	1.0e-6
CL	7.5	0.3	T3D2	30	1.0e-6
ISL	1	0.3	T3D2	26	1.0e-6
SSL	3	0.3	T3D2	23	1.0e-6

### 2.2 The establishment of different combined fusion cages and the determination of optimization parameters

**FIGURE 2:**
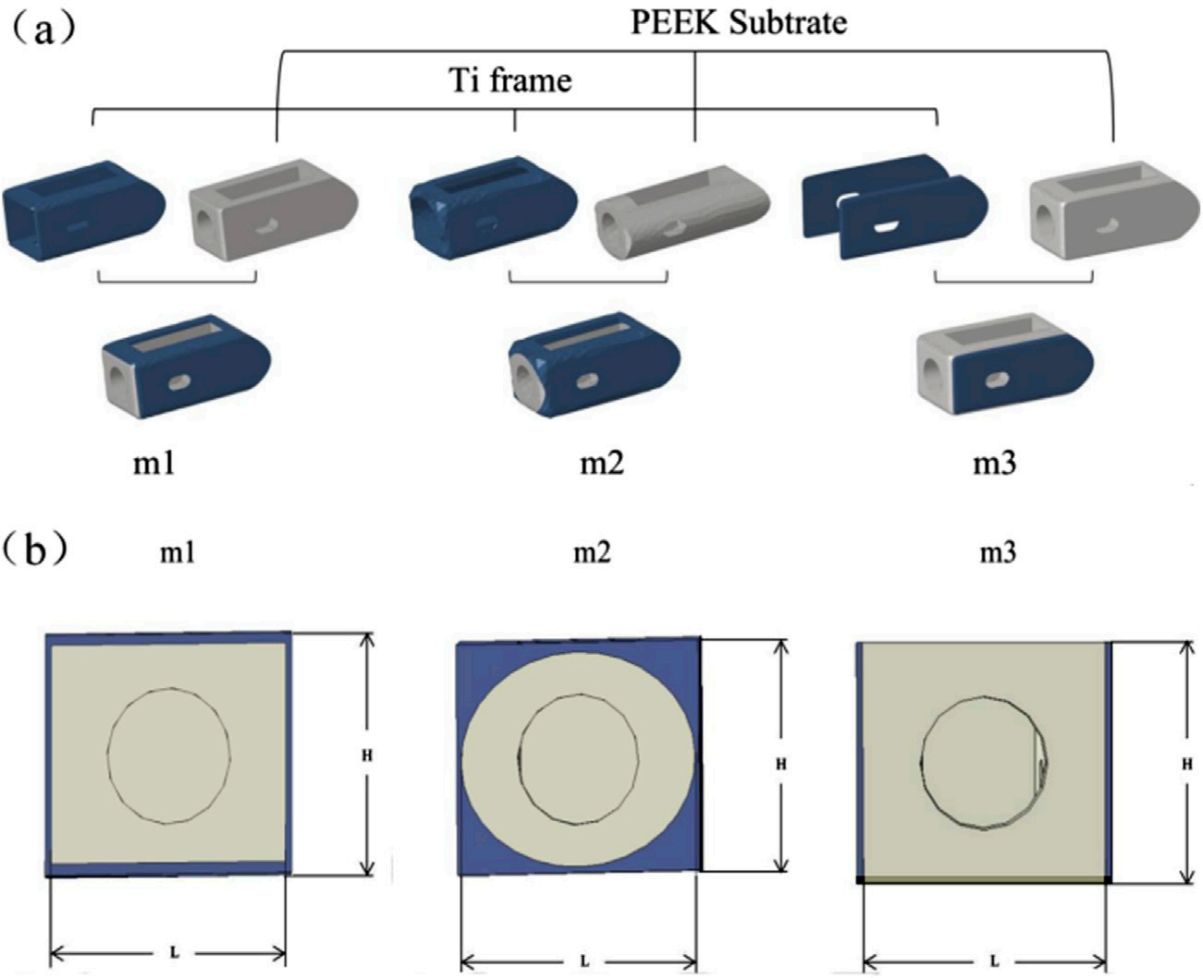
**(a)** Schematic diagram of the combinatorial cage, **(b)** Optimized Parameters Schematic Diagram.

Referring to the Hanfuser fusion device (REACH, PEEK material, Shanghai, China), three different inner core structures of the composite fusion device (m1, m2, m3) were established using the 3D modeling software Siemens NX 11.0 (Siemens, Germany), with a length of 19.3 mm, a width of 10 mm, and a height of 9 mm. The structure was divided into two parts: a titanium (Ti) frame and three different PEEK inner core structures (square hole m1, circular hole m2, and plate-m3). To eliminate potential stress concentration, the outer contour of the fusion device was rounded and smoothed. As shown in Figure 2a.

Based on the three different structures of the composite fusion devices established in the previous stage, multi-objective parameter optimization was performed on the dimensions of the composite fusion devices under the premise of ensuring perfect compatibility between the Ti frame and PEEK core interfaces of the fusion device. According to feature importance analysis (FIA), three key parameters significantly related to biomechanical performance were selected as optimization variables: Fusion device height H, PEEK core width L, and equivalent elastic modulus E of the fusion device, as shown in Figure 2b. Among these, H must adapt to variations in patient intervertebral space height (Si et al., 2020). Additionally, studies have shown that the elastic modulus of titanium alloy (110 GPa) is significantly higher than that of human cortical bone (12 GPa), posing a risk of stress shielding, where bone grafts fail to receive adequate mechanical stimulation, thereby impairing fusion outcomes (Liverani et al., 2021). Therefore, when building machine learning datasets, L was selected as the output variable, while H and E were chosen as input variables. When determining the optimization parameter constraint range, considering the limitations of the fusion device implantation process and cost, linear gradient sampling data (ΔH = 0.5 mm, ΔL = 0.5 mm) was established within the parameter space Ω = {H ∈ [8.0, 12.0] mm, L ∈ (Campbell et al., 2020; Witowski et al., 2018) mm}, yielding the correlation results between E and H, L.

### 2.3 Equivalent compression simulation of fuser

**FIGURE 3:**
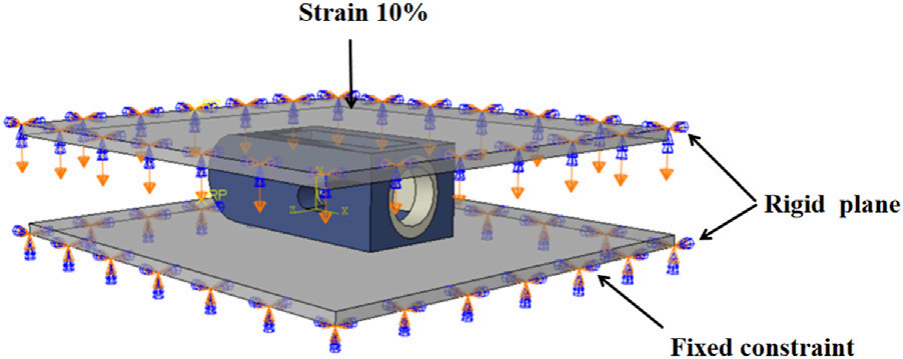
Simulated compression boundary conditions.

In order to explore the correlation between H, L and E, this study uses finite element method to simulate the compression of different structural fusion cages, as shown in Figure 3. Three-dimensional models of fusion cages with different structures are established in finite element software, and rigid load plates are established on the upper and lower surfaces of fusion cages. Tie constraint is used to realize the contact coupling between plates and fusion cages. In this study, Ti and PEEK materials are regarded as ideal elastic-plastic materials. In the elastic stage, the elastic modulus of Ti material is 110 GPa and Poisson’s ratio is 0.3, and the elastic modulus of PEEK material is 3.5 GPa and Poisson’s ratio is 0.3. In the plastic stage, the yield stress of Ti material is 944 MPa, the yield stress of PEEK material is 90.9 MPa, and the plastic strain is 0 (Feng et al., 2018). All degrees of freedom of the lower surface are fixed, and a vertical displacement load of 10% of the height is applied to the upper plate. The vertical reaction force f of the lower plate is output by the solver, and the equivalent elastic modulus e of the fusion cage is calculated according to Formula 1.

$$E=\frac{F/A}{\Delta H/H}$$ (1)

In the formula, F is the reaction force, H is the height of the fusion device, A is the effective contact area between the plate and the fusion device, and ΔH is the compression displacement.

### 2.4 Development of forecasting model

Building a machine learning model primarily involves three key steps: dataset preparation, model training, and model evaluation.

#### 2.4.1 Dataset preparation

The performance of a machine learning model is highly dependent on the quality of the training data. High-quality datasets can significantly improve the model’s accuracy and generalization ability. There are typically two ways to obtain datasets: using publicly available datasets or building your own dataset. Given that the research objective of this paper is to analyze the computational results of FE models, We chose to creat our own data through FE simulation and constructed the “H-L-E” (height-core width-equivalent elastic modulus) dataset. Among them, H and E are used as input variables, and L is used as output variable.

#### 2.4.2 Model training

**FIGURE 4:**
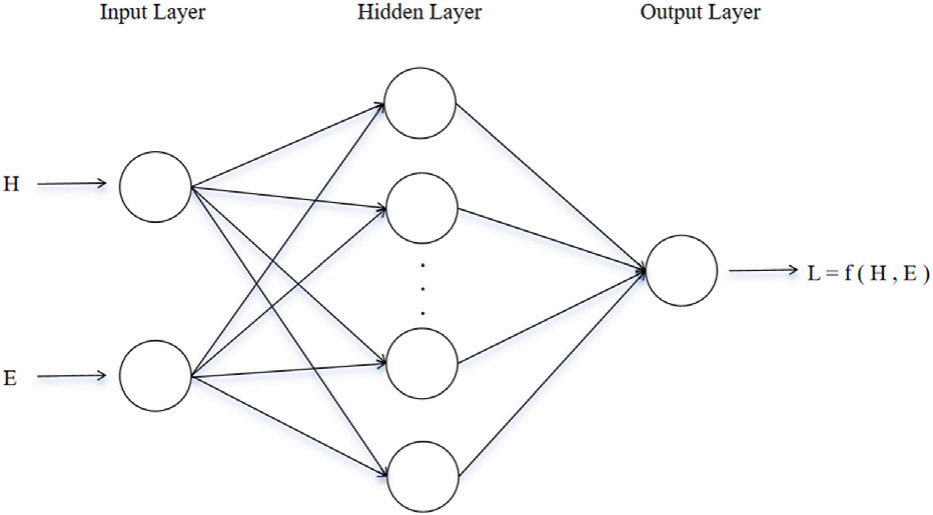
Single hidden layer BPNN structure.

Selecting an appropriate machine learning method is critical based on the nature of the research problem. The objective of this study is to predict structural parameters, making regression analysis models more suitable. A backpropagation neural network (BPNN) as shown in Figure 4 was adopted as the prediction model (Wang et al., 2015). In terms of dataset partitioning, 80% of the data is used as the training set to train the model, while the remaining 20% is used as the test set to evaluate the model’s performance. Additionally, to ensure that data with different features can meet the requirements of model training, data preprocessing is necessary. Traditional linear normalization methods are sensitive to extreme values, so this study uses standardization to preprocess the data, which exhibits greater robustness against outliers.

#### 2.4.3 Model evaluation

After model training is completed, the difference between the model’s predicted values and the actual values is measured using a loss function to evaluate the model’s performance. Commonly used performance evaluation parameters include root mean squared error (RMSE) and coefficient of determination (R^2^). RMSE and R^2^ were calculated according to Formulas 2, 3. These metrics reflect the model’s prediction accuracy and fitting performance from different perspectives.

$$RMSE=\sqrt{\frac{1}{n}\sum_{i=1}^{n}{\left(y_{i}-{\hat{y}}_{i}\right)}^{2}}$$ (2)

$$R^{2}=1-\frac{\sum_{i=1}^{n}{\left(y_{i}-{\hat{y}}_{i}\right)}^{2}}{\sum_{i=1}^{n}{\left(y_{i}-{\bar{y}}_{i}\right)}^{2}}$$ (3)

Among them, $y_{i}$ is the actual observation value, ${\hat{y}}_{i}$ is the model prediction value, and ${\bar{y}}_{i}$ is the average value of the actual observation values.

### 2.5 Multi-condition evaluation of postoperative model

**FIGURE 5:**
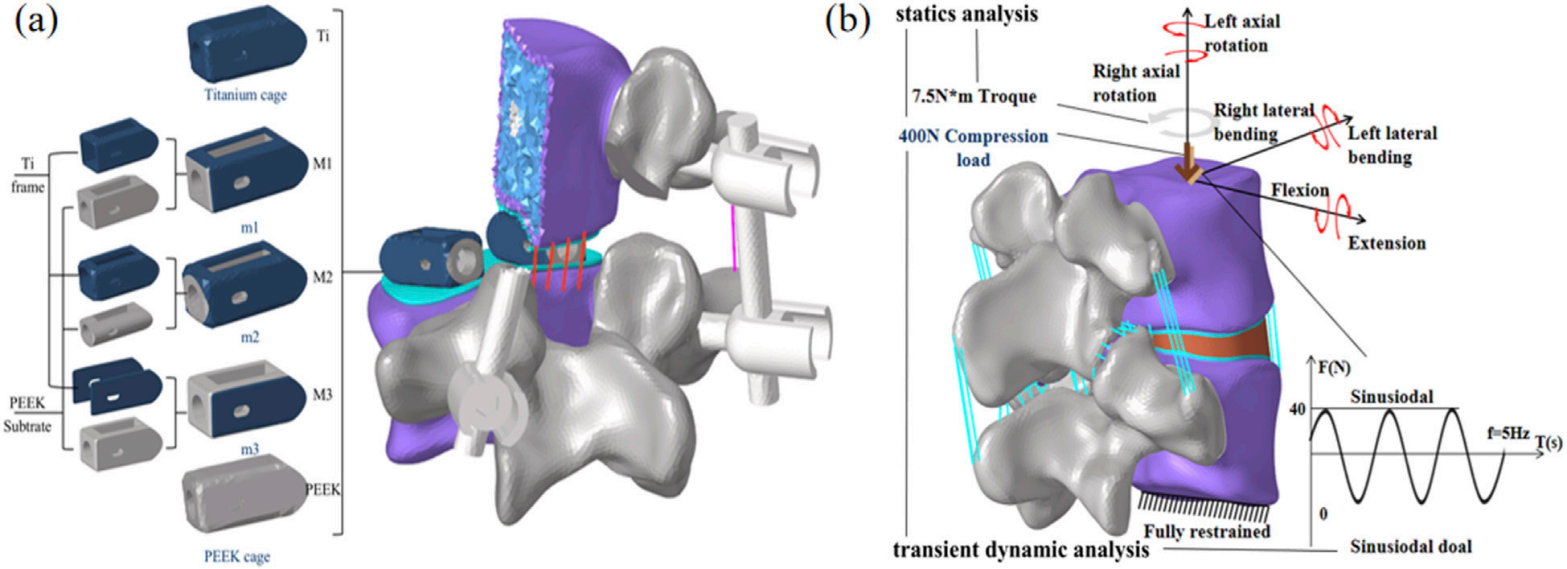
**(a)** Postoperative Model Schematic Diagram, **(b)** Loading and boundary conditions of the finite element model.

The three optimized fusion devices were implanted into a traditional PLIF postoperative model, with the Ti fusion device and PEEK fusion device serving as control groups. The modeling method and steps were the same as those described in Section of Chapter 2. The postoperative models after implantation are shown in Figure 5a.

For all finite element models, the lower surface of the L5 vertebral body is constrained. An axial preload of 400 N is applied to the upper surface of L4 to simulate corresponding physiological compression. Additionally, a torque of 7.5 N·m is applied to the upper surface of L4 to simulate six physiological states: flexion, extension, lateral bending, and axial rotation. A cyclic load of ±40 N is applied to the upper surface of L4 to simulate the mechanical environment caused by human muscle vibrations. Fusion device subsidence is one of the most common postoperative complications and is closely related to stress concentration at the upper and lower endplates. Therefore, this study focuses on analyzing and comparing the stress peaks and displacement at the upper and lower endplates; simultaneously, the stability of the postoperative model is assessed by analyzing the biomechanical response of the fixation system and postoperative range of motion; Finally, the stress shielding situation was analyzed using the biomechanical properties of the fusion device and stress energy density (ESD) to assess the force stimuli experienced during bone remodeling. The specific boundary conditions are shown in Figure 5b.

## 3 Results

### 3.1 Validation of the model

**FIGURE 6:**
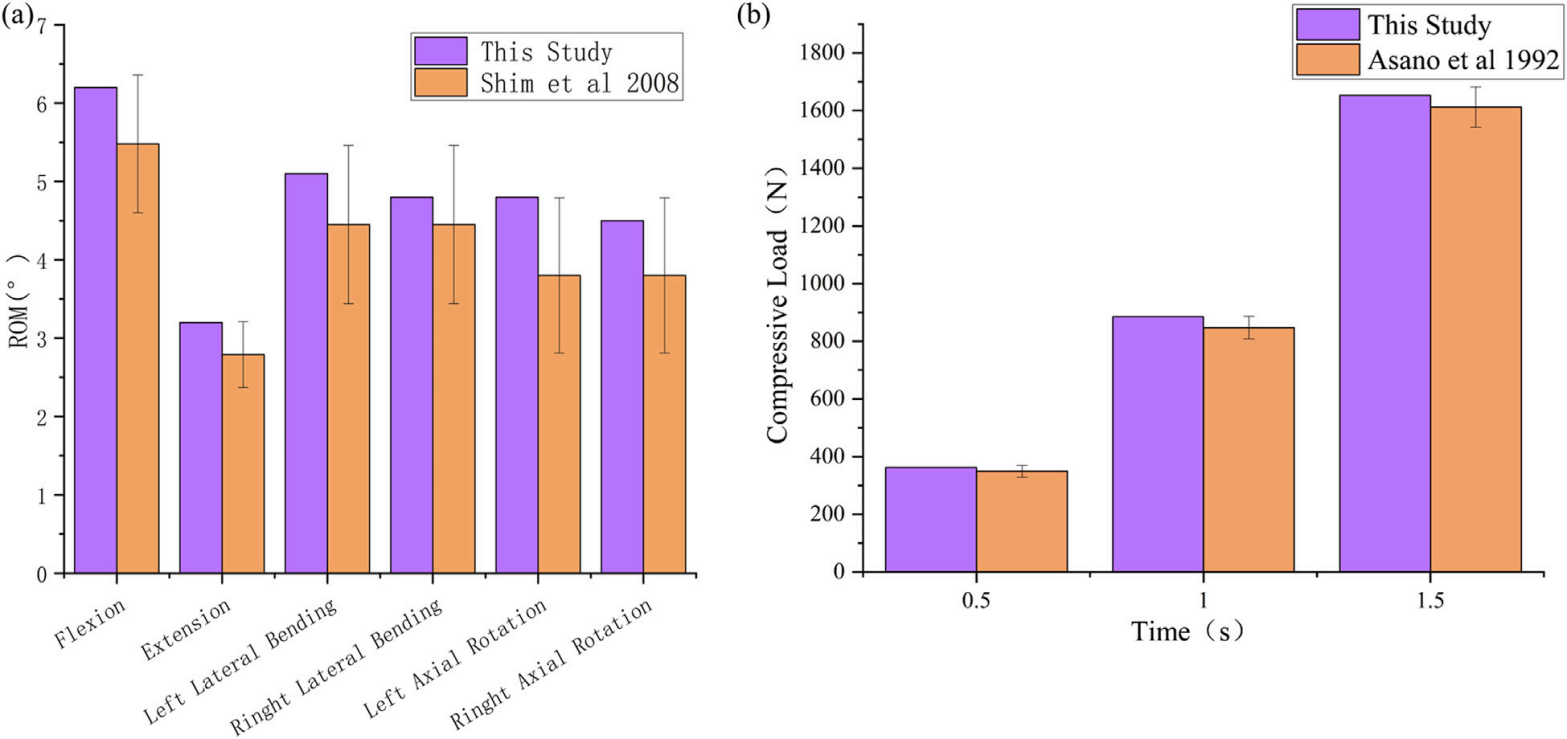
Validation of the validity of L4-L5 spinal finite element model. **(a)** ROM, **(b)** Compressive Load.

The range of motion (ROM) under six load conditions derived from the simulation analysis of the normal L4-L5 segment model was recorded and subsequently compared with the *in vitro* experimental results reported by C.S. Shim et al. (Shim et al., 2008). In this study, the ROM values obtained from the finite element model for flexion, extension, left lateral flexion, right lateral flexion, left axial rotation, and right axial rotation were 6.2, 3.2, 5.1, 4.8, 4.9, and 4.5, respectively. These results all fell within the standard deviation range of the average values obtained from *in vitro* experimental data, as shown in Figure 6a. The compression simulation validation results are shown in Figure 6b. The validity of the model was confirmed, laying the foundation for the development of subsequent surgical models.

### 3.2 Matching rate of elastic modulus of cortical bone

**FIGURE 7:**
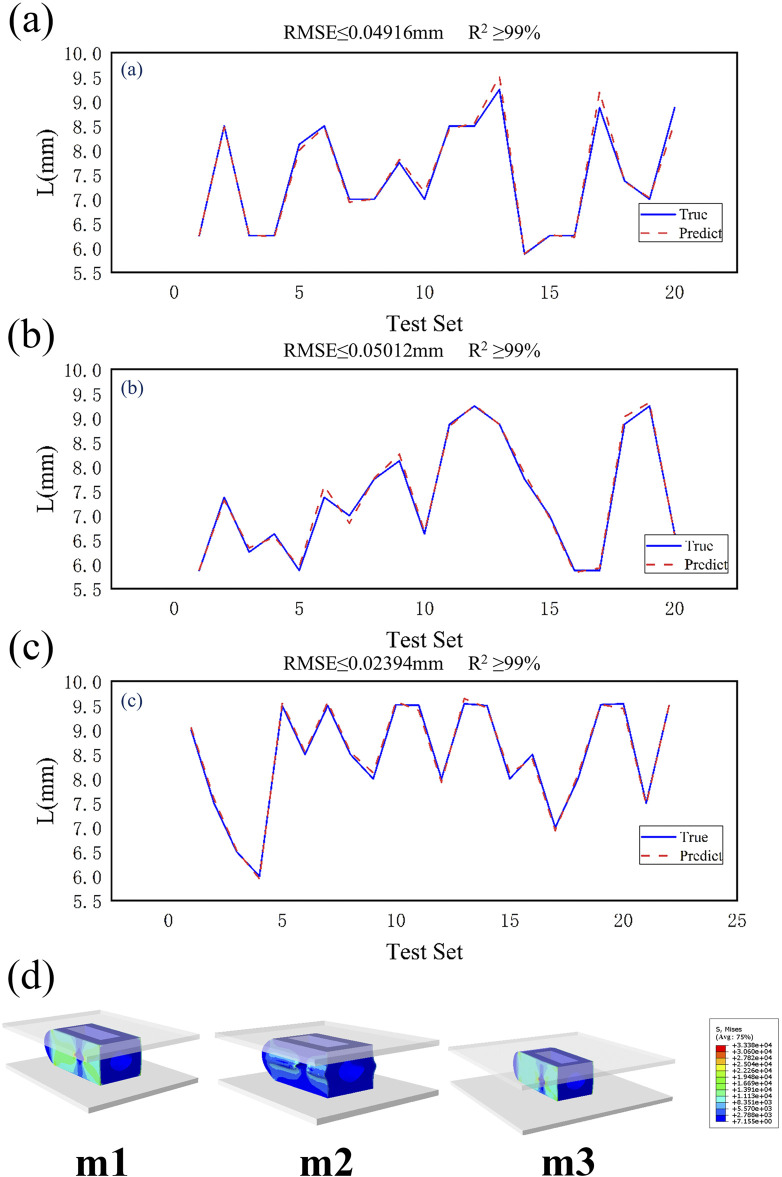
**(a)** Validation results of m1 test set, **(b)** Validation results of m2 test set, **(c)** Validation results of m3 test set, **(d)** Dimensional prediction validation contour map.

This study uses a BPNN to predict structural parameters and evaluates model performance using a 20% validation set. Two commonly used evaluation metrics, RMSE and R^2^, are employed for assessment. RMSE is the core metric for measuring prediction accuracy in BP neural networks, with smaller values indicating closer alignment between predicted and actual values. R^2^ measures the degree of variance matching between predicted and actual values, with values closer to 1 indicating greater alignment between predicted and actual values. As shown in Figures 7a–c, the RMSE values for the L values predicted by the three model sizes (m1/2/3) are relatively small compared to the actual values (0.04916 mm vs. 0.05012 mm vs. 0.02394 mm). The R^2^ values for all three models are above 99%. Considering that this study does not involve structural optimization, and the optimized size parameters are significantly correlated with the input parameters (equivalent elastic modulus E), the prediction results are relatively accurate.

Based on the prediction results, when the height H of the fusion device is 9 mm and the equivalent elastic modulus E is 12 GPa, the predicted inner core width L is shown in Table 2. Equivalent compression simulation experiments were conducted using finite element models for verification. As shown in Table 2, the equivalent elastic moduli were obtained as follows: m1 = 11.98 GPa, m2 = 12.03 GPa, and m3 = 11.99 GPa. The stress distribution contour plots for different models are shown in Figure 7d. The three optimized fusion devices were implanted into the traditional PLIF postoperative model along with the Ti fusion device and PEEK fusion device as control groups for subsequent biomechanical validation and evaluation.

**TABLE 2:** Elastic modulus prediction results.

Structure	*Height(H)*/mm	Equivalent elastic modulus(*E)*/GPa	Inner core height/mm	Inner core width(*L)*/mm	True equivalent elastic modulus/GPa
m1	9	12	8	9.512955	11.98
m2	9	12	8	9.710022	12.03
m3	9	12	9	9.543975	11.99

### 3.3 L4 biomechanical characteristics of L4 lower endplate

**FIGURE 8:**
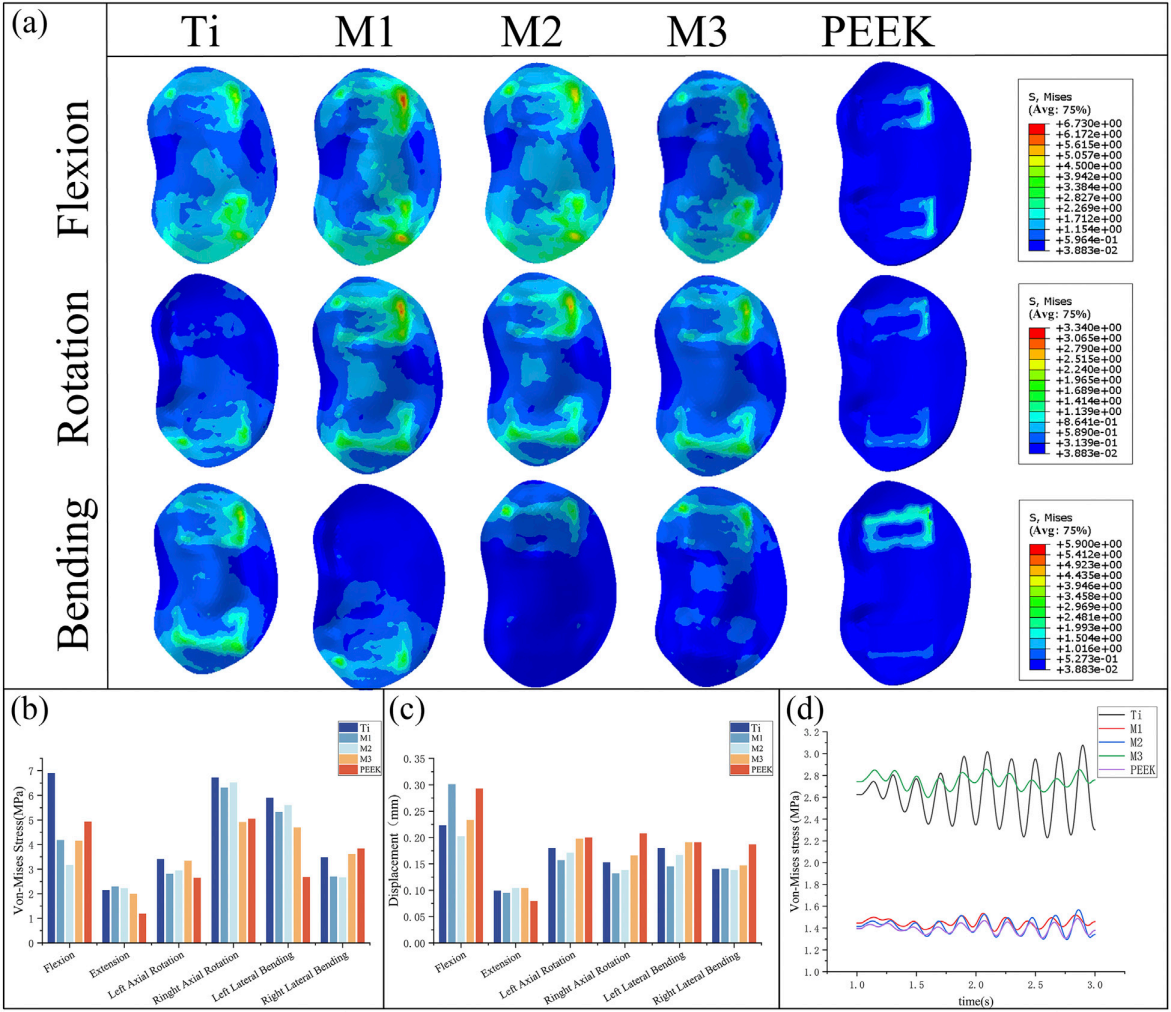
**(a)** Stress distributions of L4 lower endplates under different working conditions in five models, Comparisons of **(b)** maximum von Mises stress, **(c)** maximum displacement in the L4 lower endplate for five models at different working conditions, **(d)** Dynamic response analysis of maximum stress values on the L4 lower endplate for five models under vibration conditions.

By comparing Figures 8a,b, the peak von Mises stress and stress distribution of the L4 lower plate process under static conditions were analyzed. Under static conditions, the Ti model exhibited the highest peak von Mises stress (6.9 MPa during flexion). In contrast, the M2 model distributed stress more uniformly and demonstrated lower stress peaks, particularly under flexion and lateral bending, indicating reduced endplate stress and risk of subsidence. Under conditions such as extension, left rotation, and left bending, the PEEK model exhibited relatively lower stress values (1.18 MPa, 2.64 MPa, and 2.68 MPa). Under extension conditions, the stress peak values of all five models were relatively low. Overall, the L4 lower endplate in the Ti model exhibited relatively high stress peaks in most conditions. In the M1 model, the L4 lower plate exhibited relatively high stress peaks only in the flexion condition, while performing relatively well in other conditions with lower stress values. The M2 model exhibited relatively low stress peaks in the extension and right-bending conditions. Flexion motion is common in daily life and significantly associated with lumbar spine injury (Roman-Liu et al., 2023). M2 model exhibited the lowest peak von Mises stress on the L4 lower endplate during flexion (3.16 MPa). Compared with Ti model, the peak von Mises stress of M2 model decreased by 54.2% during flexion. The M3 model exhibits lower stress in conditions such as extension and left rotation, but stress concentration is more pronounced in right rotation and left bending conditions. The PEEK model has relatively low stress values under most conditions, but exhibits higher stress peaks in right bending conditions.

By comparing the displacement of the L4 inferior articular plate under static conditions in Figure 8c, it was found that the PEEK model had the lowest displacement value (0.079 mm) under the extension condition, while the displacement values of the PEEK model were relatively high in the other five conditions (The maximum displacement is 0.293 mm during flexion). In the flexion condition, the M1 model exhibited the highest displacement value (0.301 mm) for the L4 inferior articular process, while the M2 model had the lowest displacement value (0.202 mm). The Ti model exhibited higher displacement values in left rotation and left flexion conditions (0.18 mm), but performed better in extension, right rotation, and right flexion conditions, with lower displacement values (0.099 mm, 0.153 mm, 0.14 mm). The M1, M2, and M3 models performed similarly, exhibiting lower displacement values under conditions such as extension, left bending, and right bending, but higher displacement values under conditions such as flexion and left rotation.

Under vibration conditions, the dynamic response of the L4 endplate is shown in Figure 8d. In the M3 model, the average stress of the L4 endplate is the highest (2.748 MPa). The Ti model has the highest stress amplitude, with an average stress close to that of the M3 model (2.63 MPa), only 4.3% lower. The average stress and stress amplitude of the M1, M2, and PEEK models have similar mean stress and stress amplitude values (1.449 MPa vs. 1.416 MPa vs. 1.395 MPa), with the M1 model having the smallest stress amplitude. The instantaneous stress fluctuations in the M2 model are similar to those in the PEEK model.

### 3.4 L5 biomechanical characteristics of L5 upper endplate

**FIGURE 9:**
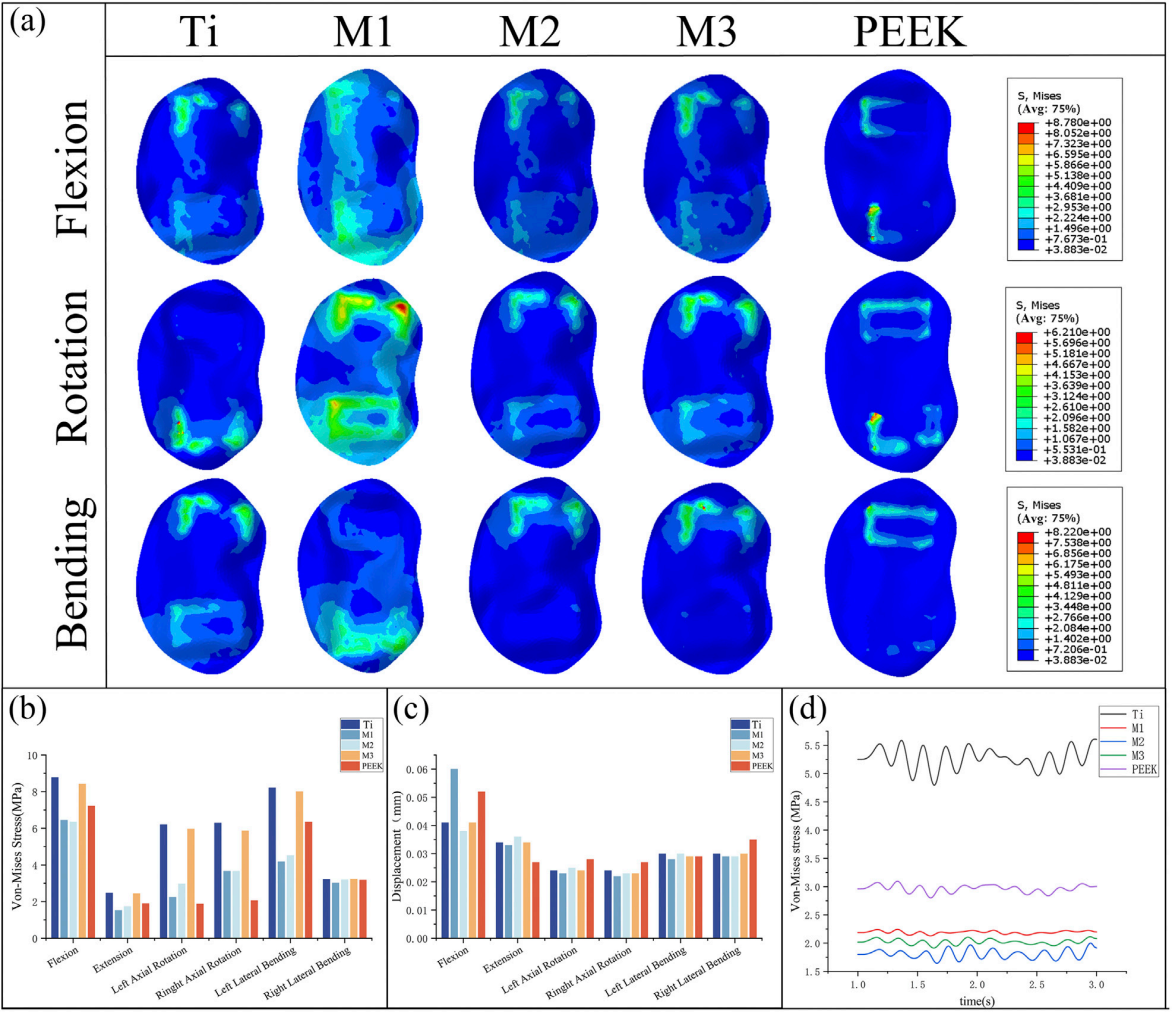
**(a)** Stress distributions of L5 upper endplates under different working conditions in five models, Comparisons of **(b)** maximum von Mises stress, **(c)** maximum displacement in the L5 upper endplate for five models at different working conditions, **(d)** Dynamic response analysis of maximum stress values on the L5 upper endplate for five models under vibration cond

By comparing Figures 9a,b, the peak von Mises stress and stress distribution of the L5 superior articular process under static conditions were analyzed. Under right-bending conditions, the peak stresses of all models were relatively close, with the M3 model exhibiting slightly higher stress (3.23 MPa) and the M1 model exhibiting relatively lower stress. Under almost all conditions, the Ti model exhibits the highest peak von Mises stress on the L5 upper endplate (8.78 MPa during flexion). The M1 model and M2 model exhibit similar performance, with lower stress peaks under conditions such as posterior extension, left rotation, and right bending, but higher stress under conditions such as anterior flexion and left bending. M2 model exhibited the lowest peak von Mises stress on the L5 upper endplate during flexion (6.35 MPa). Compared with Ti model, the peak von Mises stress of M2 model decreased by 27.7% during flexion. The M3 model exhibits high stress peaks in conditions such as flexion, left bending, right bending, and left rotation (8.43 MPa, 5.97 MPa, 5.87 MPa, 8.01 MPa), but performs well in conditions such as extension and right bending, with lower stress values (2.44 MPa, 3.23 MPa). The PEEK model exhibits higher stress in the left bending condition (7.224 MPa) but performs well in the remaining conditions.

Figure 9c compares the displacement of the L5 superior articular process under static conditions, showing that the displacement values of the L5 inferior articular process are similar across all five models in the five conditions of extension, lateral bending, and rotation. Among them, the PEEK model exhibits the lowest displacement value (0.027 mm) in the extension condition but performs poorly in the remaining conditions, with relatively high displacement values (0.035 mm during right lateral bending). In the flexion condition, the M1 model and PEEK model had the highest maximum displacement values of the L5 superior articular process (0.06 mm, 0.052 mm), while the displacement values of the other models were similar, with the M2 model having the lowest displacement value (0.038 mm). The M1, M2, and M3 models performed well in the extension, left rotation, right rotation, and left flexion conditions, with low displacement values. The M1 model had a higher displacement value in the flexion condition, while the M2 and M3 models had higher displacement values in the extension condition.

Under vibrational conditions, the dynamic response of the epiphyseal plate on L5 is shown in Figure 9d. In the Ti model, the mean stress and stress amplitude of the epiphyseal plate on L5 were the highest (5.271 MPa), followed by the PEEK model (2.965 MPa). The mean stress of the M1, M2, and M3 models show significant decreases in stress mean (2.192 MPa vs. 1.809 MPa vs. 2.023 MPa) and stress amplitude (58.4% vs. 65.7% vs. 61.6%), with the M1 model having the smallest stress amplitude and the M2 model having the smallest stress mean.

### 3.5 Biomechanical characteristics of fusion cage

**FIGURE 10:**
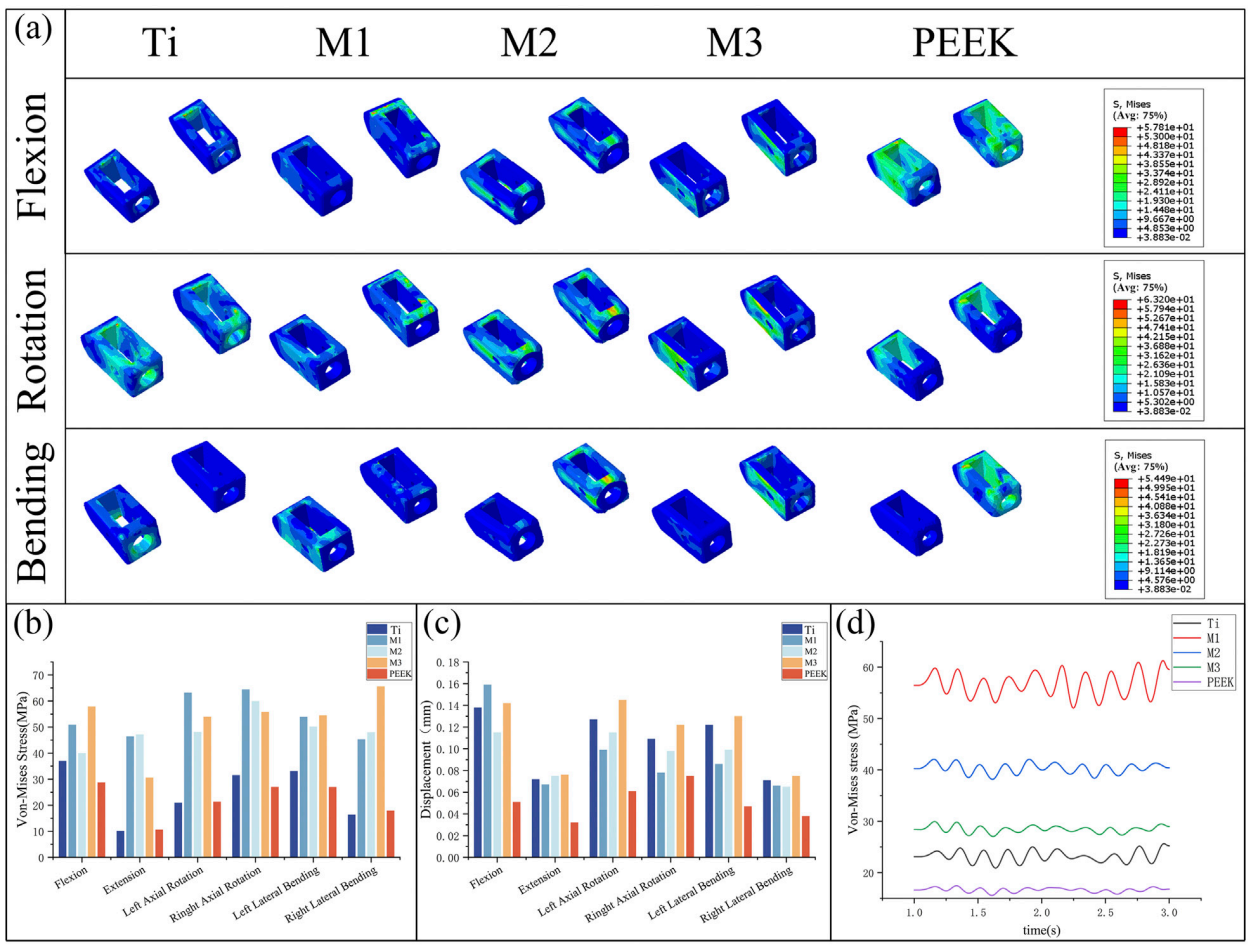
**(a)** Stress distributions of the cage under different working conditions in five models, Comparisons of **(b)** maximum von Mises stress, **(c)** maximum displacement in the cage for five models at different working conditions, **(d)** Dynamic response analysis of maximum stress values on the cage for five models under vibration conditions.

By comparing Figure 10a,b, the von Mises stress peaks and stress distributions of the coupler under static conditions were analyzed. Under all conditions, the M1, M2, and M3 models all exhibited high stress peaks in the coupler. Among these, the M1 model had higher stress peaks under left-hand and right-hand conditions (63.2 MPa, 64.39 MPa), the M3 model exhibits higher stress peaks under flexion, left bend, and right bend conditions (57.81 MPa, 54.49 MPa, 65.62 MPa), while the M2 model demonstrates the most stable stress performance among the three models, with no obvious stress concentration. The PEEK model exhibits the lowest stress peaks under conditions such as forward flexion, right rotation, and left bending (28.7 MPa, 27.05 MPa, 27.01 MPa). The Ti model exhibits the lowest stress peaks under conditions such as backward extension, left rotation, and right bending (10.14 MPa, 20.97 MPa, 16.35 MPa).

Figure 10c compares the displacement of the fusion device under static conditions. The PEEK model exhibits relatively low displacement values under all conditions (0.038 mm during extension). The Ti model and M3 model exhibit higher displacement values under all conditions, with the M3 model showing the highest displacement values under conditions such as posterior extension, lateral rotation, and lateral flexion (0.076 mm, 0.145 mm, 0.122 mm, 0.13 mm, 0.075 mm). The M2 model exhibited relatively high displacement values but all were lower than those of the M3 and Ti models. The M1 model exhibited the highest displacement value in the forward flexion condition (0.159 mm) and performed well in the remaining conditions.

Under vibration conditions, the dynamic response of the fusion device is shown in Figure 10d. The PEEK model fusion device has the lowest average stress (16.64 MPa), followed by the Ti model (23.23 MPa). The average stresses of the M1, M2, and M3 models all show a significant increase (56.71 MPa vs. 40.32 MPa vs. 28.46 MPa), with the M1 model having the highest stress amplitude.

### 3.6 Biomechanical characteristics of fixation system

**FIGURE 11:**
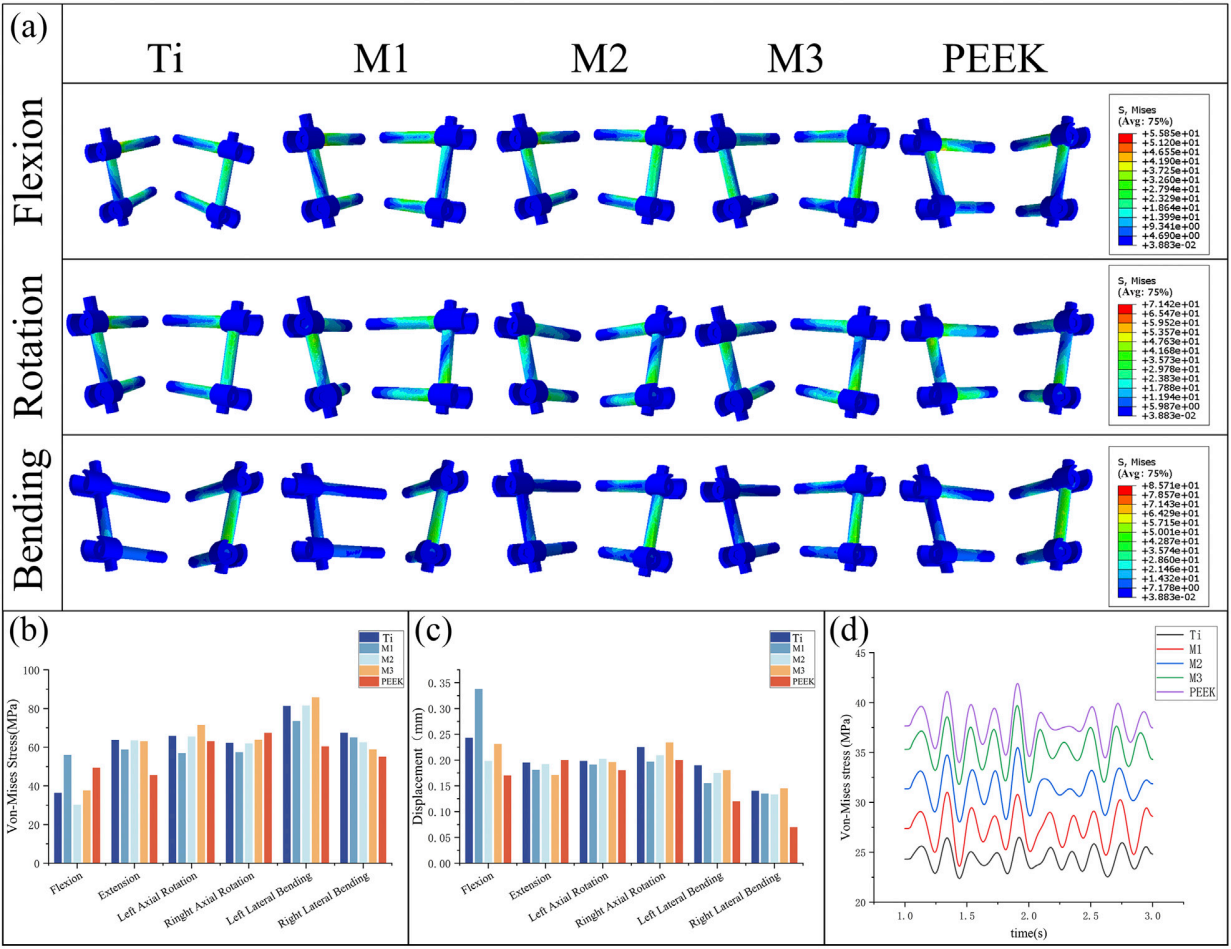
**(a)** Stress distributions of the fixation system under different working conditions in five models, Comparisons of, **(b)** maximum von Mises stress, **(c)** maximum displacement in the fixation system for five models at different working conditions, **(d)** Dynamic response analysis of maximum stress values on the fixation system for five models under vibration conditions.

By comparing Figures 11a,b, the von Mises stress peaks and stress distributions of the pedicle screw fixation system under static conditions were analyzed. Under flexion conditions, the M1 model exhibited the highest von Mises stress peak (55.95 MPa), while the M2 model had the lowest stress peak (30.22 MPa). Under other conditions, the stress peaks of the fixation systems in the five models were relatively close. Specifically, under the extension condition, the stress peaks of the Ti, M2, and M3 models were relatively high, all exceeding 63 MPa, while the stress of the PEEK model was relatively low (45.56 MPa). The Ti model exhibits higher stress peaks under left flexion and right flexion conditions, while the M1 model has higher stress peaks under flexion conditions but performs relatively well under other conditions with lower stress values. The M2 model has higher stress peaks under extension and left rotation conditions but lower stress under flexion conditions. The M3 model exhibits significantly higher stress peaks under left rotation and left flexion conditions compared to other models.

Figure 11c compares the displacement of the pedicle screw fixation system under static conditions. Under flexion conditions, the M1 model has the highest maximum displacement value (0.338 mm), while the PEEK model has the lowest displacement value (0.17 mm). In the remaining five conditions, the maximum displacement values of the fixation systems in different models were relatively close, with the PEEK model exhibiting the best displacement performance in left rotation, left bending, and right bending conditions (0.18 mm, 0.12 mm, and 0.07 mm, respectively). The M3 model exhibited the largest displacement of the fixed system under right rotation and right bending conditions (0.234 mm, 0.145 mm), while under posterior extension conditions, the displacement of the M3 model was relatively low (0.171 mm). The M2 model performed stably under all conditions, with moderate displacement values.

Under vibrational conditions, the dynamic response of the pedicle screw fixation system is shown in Figure 11d. The transient stress fluctuations of the five models are similar. Among these, the PEEK model exhibits the highest average stress (37.72 MPa), while the Ti model has the lowest average stress (24.39 MPa). The stress amplitudes of the M1, M2, and M3 models are similar to those of the PEEK model, with average stresses falling between the two (27.48 MPa vs. 31.43 MPa vs. 35.38 MPa).

### 3.7 Stability of postoperative model

**FIGURE 12:**
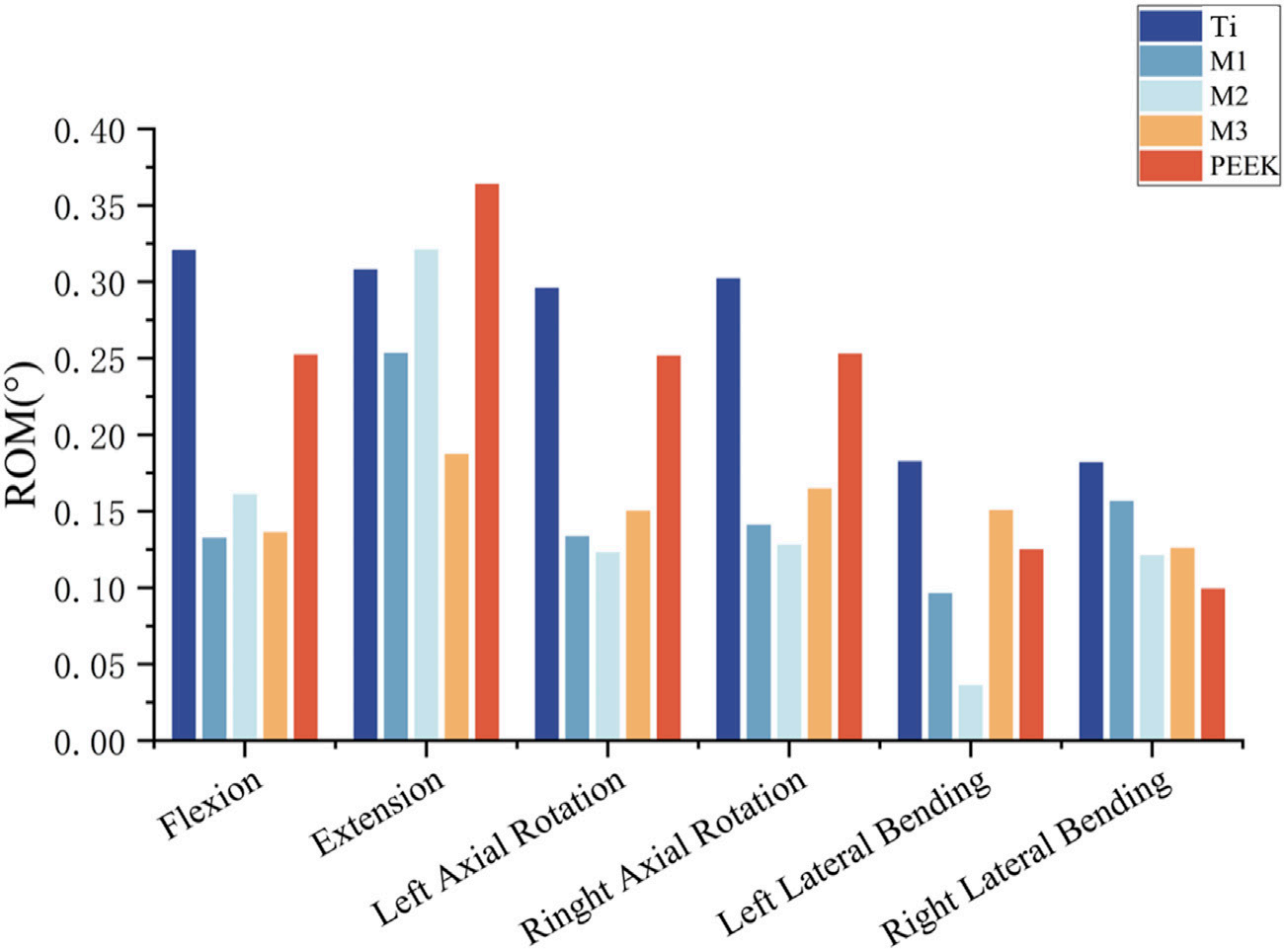
The ROM statistics of five models under static analysis.

To analyze and compare the stabilizing effects of five different fusion devices on the spine after implantation, the vertebral mobility of the five models was recorded under six different working conditions. For the Ti model, the vertebral mobility under flexion, extension, left rotation, right rotation, left flexion, and right flexion were 0.3206°, 0.3081°, 2959°, 0.3022°, 0.1826°, and 0.182°, respectively; The vertebral motion in the flexion, extension, left rotation, right rotation, left flexion, and right flexion states for the M1 model were: 0.1326°, 0.2534°, 0.1336°, 0.1411°, 0.0963°, and 0.1567°, respectively; The vertebral mobility of the M2 model under flexion, extension, left rotation, right rotation, left bending, and right bending conditions are as follows: 0.161°, 0.321°, 0.132°, 0.128°, 0.036°, and 0.121°; The vertebral mobility of the M3 model under flexion, extension, left rotation, right rotation, left bending, and right bending conditions are as follows: 0.1362°, 0.1873°, 0.1502°, 0.1648°, 0.1507°, and 0.126°; The vertebral mobility of the PEEK model under flexion, extension, left rotation, right rotation, left bending, and right bending conditions are as follows: 0.2523°, 0.364°, 0.2517°, 0.2529°, 0.125°, and 0.0993°, respectively. For detailed information, please refer to Figure 12.

### 3.8 Strain energy analysis

**FIGURE 13:**
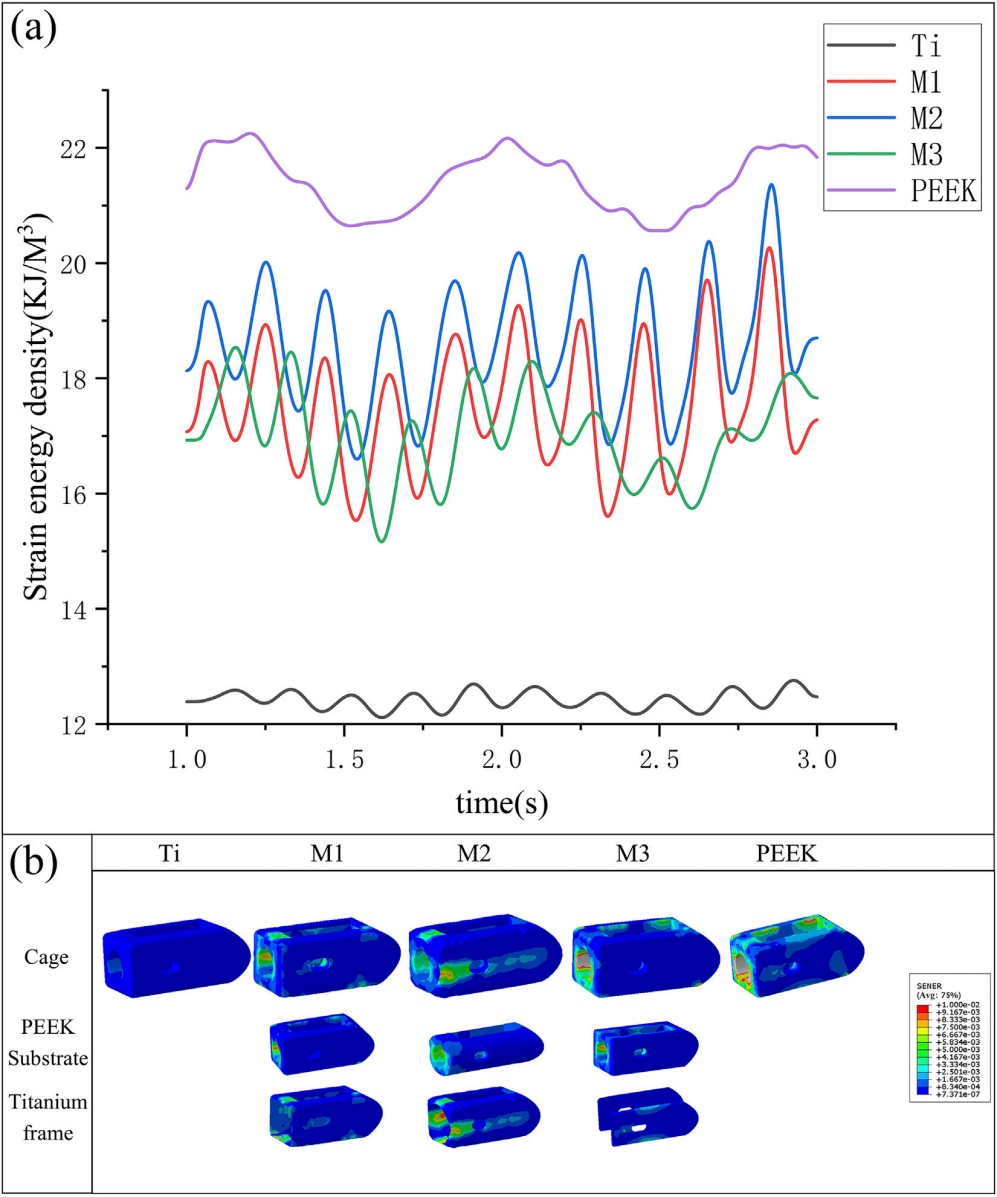
**(a)** Variation in strain energy density of fusion cages across five models under vibration conditions, **(b)** Variation of strain energy density in different fusion devices under vibration conditions.

Under vibrational conditions, the dynamic response of strain energy within the surgical model is shown in Figure 13a. The PEEK model exhibits the highest strain energy (21.43 kJ/m^3^). The Ti model has a smaller amplitude of vibration and the lowest strain energy (12.42 kJ/m^3^). The strain energies of the M1, M2, and M3 models (17.51 kJ/m^3^ vs. 18.59 kJ/m^3^ vs. 17.02 kJ/m^3^) fall between the two, with the M1 and M2 models exhibiting stable vibration and the largest amplitude of vibration. Compared with the Ti model, the strain energy of the M2 model increased by 49.7%.

The strain energy density contour plots of different fusion devices under transient dynamics analysis are shown in Figure 13b. The PEEK fusion device exhibits the highest strain energy density, concentrated around the bone graft window and observation window; the titanium alloy fusion device has the lowest strain energy density; The strain energy densities of all three optimized fusion devices are significantly higher than that of the titanium alloy fusion device. Among them, the circular hole type fusion device used in the M2 model has the largest strain energy density and the most uniform distribution. The strain energy density distribution on the titanium alloy frame and PEEK core indicates its outstanding performance in load transfer and shock absorption.

## 4 Discussion

This study established three types of combination fusion devices with equivalent elastic moduli close to cortical bone for different intervertebral space heights through a combination of biomechanical dimensional optimization and machine learning prediction. These devices were implanted into a classic PLIF surgical model, and a control group was established using a PLIF surgical model with implanted titanium alloy fusion devices and PEEK fusion devices. Using finite element analysis, the study simulated the static biomechanical states and whole-body vibration conditions of the L4-L5 segment post-lumbar fusion surgery. In the static analysis, the biomechanical characteristics of different regions were compared under six conditions—flexion, extension, lateral flexion, rotation, and rotation in both directions—after implantation of the five fusion devices, while also assessing the overall stability of the model. In the transient dynamic analysis, the dynamic stress responses and strain energy dynamic responses of different regions under whole body vibration loads were compared. The study found that compared to the Ti fusion device, the three combination fusion devices significantly reduced stress shielding, lowered stress on the bony endplates, and reduced the risk of endplate damage, collapse, and fusion device settlement; Compared to the PEEK fusion device, the combination fusion devices reduced endplate displacement, improved model stability, and the titanium alloy surface of the combination fusion devices exhibited good biocompatibility, promoting bone ingrowth and enhancing postoperative fusion outcomes; Among the three combination fusion devices, the M2 model using the circular hole type fusion device demonstrated excellent performance in the endplate region under dynamic loads, offering superior fusion outcomes in minimally invasive procedures with limited fixation ranges (Dua et al., 2020).

Among postoperative complications in lumbar fusion surgery, fusion cage subsidence is one of the primary causes of fusion failure (Yao et al., 2020). Persistent spinal pain syndrome (PSPS), a term introduced by the International Association for the Study of Pain, describes chronic or recurrent spinal pain and supersedes older, potentially stigmatizing terms like ‘failed back surgery’ (Eric and Robert, 2022). The primary cause of fusion cage subsidence is stress concentration at the bone-implant interface, which can damage the relatively fragile endplates and lead to cage subsidence. Based on previous research findings, endplate stress can serve as an indicator of challenges related to cage subsidence. Therefore, studying the bony endplates in postoperative models is particularly important. In endplate stress analysis, the biomechanical responses of different optimized fusion cage postoperative models under various conditions are compared and analyzed. Under most conditions, titanium alloy fusion devices result in significant stress concentration at the endplates, while PEEK fusion devices exhibit lower endplate stress post-implantation. This is related to the significantly higher elastic modulus of titanium alloy compared to cortical bone. Although high stiffness provides excellent compressive strength, it also leads to excessive stress concentration at the bone-implant interface; the M2 model significantly reduces stress peaks under conditions such as flexion and lateral bending. The circular hole type inner core design uniformly distributes the load, enabling better load transmission. Under vibration conditions, the stress response curve of the endplate in the Ti model clearly exhibits excessively high stress averages and more severe amplitude fluctuations. The other four models show improvements. The PEEK inner core can better achieve vibration damping effects. However, the M3 model performs poorly on the upper bony endplate. Analysis indicates that the clamp-type design used in the M3 model, although its overall equivalent elastic modulus is close to that of cortical bone, results in the PEEK inner core bearing minimal actual load during use. The reduced contact area leads to stress concentration and fails to leverage the vibration damping properties of PEEK material. However, the fusion surfaces of the M1 and M2 fusion devices that contact the endplate are made of titanium alloy. This not only leverages the excellent biocompatibility of titanium alloy but also uniformly distributes loads, transmitting them to the PEEK core, effectively reducing the impact of muscle vibrations on fusion outcomes (Fan and Guo, 2021).

This study analyzes and compares the biomechanical responses of fusion devices, taking into account that the M1/2/3 models are composite material designs. At the interfaces between different materials, significant shear forces are generated during load transmission, leading to stress concentration, which may affect the service life of the fusion devices (Wang et al., 2019). The analysis found that the internal stresses in the Ti and PEEK models are relatively small, while the internal stress in the M1 model is significantly higher than in the other four models. Under vibrational conditions, the M1 model also exhibits the largest amplitude fluctuations. Analysis reveals that stress concentration occurs at the interface between the rectangular inner core and outer shell, particularly at the sharp corners, which may cause interface deformation, inner core loosening, and fusion failure. In future studies, optimization schemes such as rounded chamfers should be considered; The M3 model performs well, as the efficiency of load transfer from the titanium alloy plates to the PEEK inner core is relatively low; The M2 model exhibits relatively stable dynamic stress response. Although it moderately increases stress within the fusion device, it remains significantly below the yield strength of PEEK material (120–150 MPa) and titanium alloy (800–950 MPa). Previous studies have shown that increasing the mechanical stress stimulus at the bone-implant interface can improve postoperative bone fusion (Huang et al., 2023).

This study evaluated postoperative model stability by comparing the biomechanical responses of pedicle screw fixation systems and postoperative model mobility. Comparative analysis revealed significant stress differences among the five surgical models under flexion and extension conditions, while only minor differences were observed under lateral rotation and lateral conditions. This is because traditional PLIF surgical models use two symmetrical fusion devices on either side, providing sufficient support and stability during physiological activities with significant lateral differences. In transient dynamic analysis, the stress amplitude and fluctuation patterns of the five models were similar. Analysis revealed that the fixation system bore the primary load under vibration conditions, with stress fluctuations being most sensitive to load changes. Analysis of postoperative model mobility indicated that all three fusion device combinations could enhance postoperative model stability to some extent. This is because all three fusion devices have been optimized through predictive modeling, resulting in equivalent elastic moduli that closely match those of cortical bone. Within the L4-L5 lumbar vertebral segment, the stiffness of the fusion cage is best matched to that of the cortical bone of the adjacent vertebral bodies, which primarily bear the load. This design avoids the high stiffness of titanium alloy fusion devices causing damage to the endplates and prevents the deformation of PEEK fusion devices from affecting changes in intervertebral disc height (Bataineh and Janaideh, 2019).

Strain energy is the energy stored in a material during deformation under external forces. It reflects the material’s ability to store and convert energy during deformation. When an external force acts on an object, the object deforms, and the work done by the external force is converted into energy stored within the object, which is strain energy. As the external force gradually decreases, the deformation decreases, and the solid releases part of the stored energy to perform work, which is the elastic deformation energy. In biomechanical analysis, this parameter is commonly used to assess stress shielding risks and has been widely applied in algorithms for bone reconstruction and tissue differentiation (Lu and Lu, 2019). This study analyzed and compared the fluctuations in strain energy density under vibrational conditions for five models, finding that the strain energy density in the Ti model was significantly lower than that in the other four models (Parisien et al., 2022). the strain energy density in the PEEK model was relatively high, and the strain energy density in the M1/2/3 models was intermediate between the two. Among these, the M2 model, which uses a circular hole type internal core fusion device, had a higher strain energy density and exhibited more sensitive fluctuations. This indicates that these fusion devices, especially the circular hole type fusion device, can promote bone ingrowth and improve fusion outcomes more effectively compared to the titanium alloy fusion device.

This study introduced a novel design framework for composite fusion devices, leveraging machine learning and finite element analysis to achieve biomechanical performance akin to cortical bone. Among the designs, the circular hole M2 model demonstrated the best overall performance, offering a promising solution for reducing stress shielding and improving spinal fusion outcomes. This study also has certain limitations. Three different structures were created for analysis and comparison, making it impossible to optimize the structure and material ratios to the fullest extent. In subsequent studies, topological optimization will be incorporated to identify the optimal structure; Additionally, to simplify computational complexity, the geometric structure of the fusion device was simplified during parametric modeling, such as by fixing the thickness of the upper and lower layers of the titanium alloy to 0.5 mm to match the thickness of the bony endplates. The impact of titanium alloy thickness on fusion outcomes should be explored in future studies, and changes in endplate thickness in patients with lumbar degenerative diseases should also be considered; This study only considered two materials: titanium alloy and PEEK. Future research may introduce other materials with better performance for optimization; When optimizing structural parameters based on patient physiological data, this study only considered cortical bone elastic modulus and intervertebral disc height. In future studies, the parameter range should be expanded to find a more patient-specific fusion device structure that better adapts to the patient’s physiological state.

## 5 Conclusion

This study utilized three-dimensional modeling software to establish three different types of frame-type composite structure fusion devices. For each structure, the structural ratio equivalent to the elastic modulus of cortical bone was predicted using a backpropagation neural network (BPNN). The three composite fusion devices were implanted into a traditional PLIF postoperative model alongside a titanium alloy fusion device and a PEEK fusion device as control groups, and static and transient dynamic analyses were conducted to compare the biomechanical responses of the lumbar spine at various locations following implantation of the five fusion devices. The results indicate that all three optimized fusion devices effectively reduce the risk of fusion device settlement, mitigate stress shielding effects, improve fusion rates, and enhance postoperative model stability, with the circular hole inner core fusion device performing the best. This study introduces a new method for personalized fusion device design: “experimental finite element simulation-machine learning prediction-postoperative model biomechanical validation and evaluation.” This approach can effectively reduce the design cycle and cost of personalized orthopedic implants, laying the foundation for designing fusion devices tailored to patients’ physiological conditions.

## Data Availability

The original contributions presented in the study are included in the article/supplementary material, further inquiries can be directed to the corresponding authors.

## References

[B1] Al-dulimi Z. Wallis M. Tan D. K. Maniruzzaman M. Nokhodchi A. (2021). 3D printing technology as innovative solutions for biomedical applications. Drug Discov. Today 26 (2), 360–383. 10.1016/j.drudis.2020.11.013 33212234

[B2] Ayturk U. M. Puttlitz C. M. (2011). Parametric convergence sensitivity and validation of a finite element model of the human lumbar spine. Comput. Methods Biomechanics Biomed. Eng. 14 (8), 695–705. 10.1080/10255842.2010.493517 21229413

[B3] Bataineh K. Janaideh A. M. (2019). Effect of different biocompatible implant materials on the mechanical stability of dental implants under excessive oblique load. Clin. Implant Dent. Relat. Res. 21 (6), 1206–1217. 10.1111/cid.12858 31670872

[B4] Campbell P. G. Cavanaugh D. A. Nunley P. Utter P. A. Kerr E. Wadhwa R. (2020). PEEK versus titanium cages in lateral lumbar interbody fusion: a comparative analysis of subsidence. Neurosurg. Focus 49 (3), e10. 10.3171/2020.6.focus20367 32871573

[B5] Duan K. Qin Y. Ye J. Zhang W. Hu X. Zhou J. (2020). Percutaneous endoscopic debridement with percutaneous pedicle screw fixation for lumbar pyogenic spondylodiscitis: a preliminary study. Int. Orthop. 44 (3), 495–502. 10.1007/s00264-019-04456-1 31879810 PMC7026210

[B6] Eric C.-Pu C. Robert J. T. (2022). Effectiveness of multimodal chiropractic care featuring spinal manipulation for persistent spinal pain syndrome following lumbar spine surgery: retrospective chart review of 31 adults in Hong Kong. Med. Sci. Monit. 28, e937640. 10.12659/msm.937640 35915570 PMC9357349

[B7] Fan W. Guo L. X. (2021). Prediction of the influence of vertical whole-body vibration on biomechanics of spinal segments after lumbar interbody fusion surgery. Clin. Biomech. 86, 105389. 10.1016/j.clinbiomech.2021.105389 34052692

[B8] Fan W. Guo L. X. Zhao D. (2021). Posterior lumbar interbody fusion *versus* transforaminal lumbar interbody fusion: finite element analysis of the vibration characteristics of fused lumbar spine. World Neurosurg. 150, e81–e88. 10.1016/j.wneu.2021.02.094 33647495

[B9] Feng Q. Tang Q. Liu Y. Setchi R. Soe S. Ma S. (2018). Quasi-static analysis of mechanical properties of Ti6Al4V lattice structures manufactured using selective laser melting. Int. J. Adv. Manuf. Technol. 94 (5-8), 2301–2313. 10.1007/s00170-017-0932-7

[B10] Hasegawa T. Ushirozako H. Shigeto E. Ohba T. Oba H. Mukaiyama K. (2020). The titanium-coated PEEK cage maintains better bone fusion with the endplate than the PEEK cage 6 months after PLIF surgery: a multicenter, prospective, randomized study. Spine 45 (15), e892–E902. 10.1097/brs.0000000000003464 32675599

[B11] Hasler C. C. (2013). A brief overview of 100 years of history of surgical treatment for adolescent idiopathic scoliosis. J. Children’s Orthop. 7 (1), 57–62. 10.1007/s11832-012-0466-3 24432060 PMC3566253

[B12] Huang S. F. Chang C. M. Liao C. Y. Chan Y. T. Li Z. Y. Lin C. L. (2023). Biomechanical evaluation of an osteoporotic anatomical 3D printed posterior lumbar interbody fusion cage with internal lattice design based on weighted topology optimization. Int. J. Bioprinting 9 (3), 697. 10.18063/ijb.697 37273986 PMC10236481

[B13] Kafle A. Luis E. Silwal R. Pan H. M. Shrestha P. L. Bastola A. K. (2021). 3D/4D printing of polymers: fused deposition modelling (FDM), selective laser sintering (SLS), and stereolithography (SLA). Polymers 13 (18), 3101. 10.3390/polym13183101 34578002 PMC8470301

[B14] Lee J. J. Jacome F. P. Hiltzik D. M. Pagadala M. S. Hsu W. K. (2024). Evolution of titanium interbody cages and current uses of 3D printed titanium in spine fusion surgery. Curr. Rev. Musculoskelet. Med. 18, 635–644. 10.1007/s12178-024-09912-z 39003679 PMC12446159

[B15] Liverani E. Rogati G. Pagani S. Brogini S. Fortunato A. Caravaggi P. (2021). Mechanical interaction between additive-manufactured metal lattice structures and bone in compression: implications for stress shielding of orthopaedic implants. J. Mech. Behav. Biomed. Mater. 121, 104608. 10.1016/j.jmbbm.2021.104608 34077904

[B16] Lu T. Lu Y. (2019). Comparison of biomechanical performance among posterolateral fusion and transforaminal, extreme, and oblique lumbar interbody fusion: a finite element analysis. World Neurosurg. 129, e890–e899. 10.1016/j.wneu.2019.06.074 31226452

[B17] Makino T. Takaneka S. Sakai Y. Yoshikawa H. Kaito T. (2021). Impact of mechanical stability on the progress of bone ongrowth on the frame surfaces of a titanium-coated PEEK cage and a 3D porous titanium alloy cage: *in vivo* analysis using CT color mapping. Eur. Spine J. 30 (5), 1303–1313. 10.1007/s00586-020-06673-4 33389201

[B18] Murphy M. P. Brown N. M. (2021). CORR synthesis: when should the orthopaedic surgeon use artificial intelligence, machine learning, and deep learning. Clin. Orthop. Relat. Res. 479 (7), 1497–1505. 10.1097/corr.0000000000001679 33595930 PMC8208440

[B19] Parisien A. Wai E. K. Elsayed M. S. A. Frei H. (2022). Subsidence of spinal fusion cages: a systematic review. Int. J. Spine Surg. 16 (6), 1103–1118. 10.14444/8363 36289005 PMC9807049

[B20] Polikeit A. Ferguson S. J. Nolte L. P. Orr T. E. (2003). Factors influencing stresses in the lumbar spine after the insertion of intervertebral cages: finite element analysis. Eur. Spine J. 12 (4), 413–420. 10.1007/s00586-002-0505-8 12955610 PMC3467788

[B21] Roman-Liu D. Kamińska J. Tokarski T. (2023). Differences in lumbar spine intradiscal pressure between standing and sitting postures: a comprehensive literature review. PeerJ 11, e16176. 10.7717/peerj.16176 37872945 PMC10590571

[B22] Seifi M. Gorelik M. Waller J. Hrabe N. Shamsaei N. Daniewicz S. (2017). Progress towards metal additive manufacturing standardization to support qualification and certification. JOM 69 (3), 439–455. 10.1007/s11837-017-2265-2

[B23] Shim C. S. Park S. W. Lee S.-H. Lim T. J. Chun K. Kim D. H. (2008). Biomechanical evaluation of an interspinous stabilizing device, locker. Spine 33 (22), e820–e827. 10.1097/brs.0b013e3181894fb1 18923305

[B24] Si G. Li T. Liu X. Liu Z. Li W. Yu M. (2020). Correlation analysis between postoperative hip pain and spino-pelvic/hip parameters in adult scoliosis patients after long-segment spinal fusion. Eur. Spine J. 29 (12), 2990–2997. 10.1007/s00586-020-06316-8 32006111

[B25] Tan J. H. Cheong C. K. Hey H. W. D. (2021). Titanium (ti) cages May be superior to polyetheretherketone (PEEK) cages in lumbar interbody fusion: a systematic review and meta-analysis of clinical and radiological outcomes of spinal interbody fusions using Ti *versus* PEEK cages. Eur. Spine J. 30 (5), 1285–1295. 10.1007/s00586-021-06748-w 33555365

[B26] Verma S. Sharma N. Kango S. Sharma S. (2021). Developments of PEEK (Polyetheretherketone) as a biomedical material: a focused review. Eur. Polym. J. 147, 110295. 10.1016/j.eurpolymj.2021.110295

[B27] Wang L. Zeng Y. Chen T. (2015). Back propagation neural network with adaptive differential evolution algorithm for time series forecasting. Expert Syst. Appl. 42 (2), 855–863. 10.1016/j.eswa.2014.08.018

[B28] Wang Y. Wang M. Yin K. Huang A. h. Li Y. s. Huang C. x. (2019). Yielding and fracture behaviors of coarse-grain/ultrafine-grain heterogeneous-structured copper with transitional interface. Trans. Nonferrous Metals Soc. China 29 (3), 588–594. 10.1016/s1003-6326(19)64967-8

[B29] Wellington I. J. Kia C. Coskun E. Torre B. B. Antonacci C. L. Mancini M. R. (2022). Cervical and lumbar disc arthroplasty: a review of current implant design and outcomes. BIOENGINEERING-BASEL 9 (5), 227. 10.3390/bioengineering9050227 35621505 PMC9137579

[B30] Witowski J. Sitkowski M. Zuzak T. Coles-Black J. Chuen J. Major P. (2018). From ideas to long-term studies: 3D printing clinical trials review. Int. J. Comput. Assisted Radiology Surg. 13 (9), 1473–1478. 10.1007/s11548-018-1793-8 29790077 PMC6132399

[B31] Yan Y. Yu J. Wang Y. Dong H. Zhang K. Wang Y. (2023). A newly designed personalized interbody fusion cage and its biomechanical analysis. Acta Mech. Sin. 39 (9), 623047. 10.1007/s10409-023-23047-x

[B32] Yao Y. C. Chou P. H. Lin H. H. Wang S. T. Liu C. L. Chang M. C. (2020). Risk factors of cage subsidence in patients received minimally invasive transforaminal lumbar interbody fusion. Spine 45 (19), e1279–E1285. 10.1097/brs.0000000000003557 32472823

[B33] Zhang W. Sun C. Zhu J. Leng H. Song C. (2020). 3D printed porous titanium cages filled with simvastatin hydrogel promotes bone ingrowth and spinal fusion in rhesus macaques. Biomaterials Sci. 8 (15), 4147–4156. 10.1039/d0bm00361a 32496502

